# Mediterranean Diet Adherence, Weight Status, and Self-Concept in Primary Schoolchildren in Melilla, Spain: A Comparison of Two Independent Cohorts Assessed in 2018 and 2024

**DOI:** 10.3390/healthcare14213697

**Published:** 2026-11-01

**Authors:** Guillermo Moreno-Rosa, Manuel Castro-Sánchez

**Affiliations:** Department of Didactics of Musical, Plastic and Corporal Expression, Faculty of Education and Sports Sciences of Melilla, University of Granada, Santander Av. 1, 52071 Melilla, Spain; manuelcs@ugr.es

**Keywords:** body mass index, mediterranean diet, primary schoolchildren, self-concept, weight status

## Abstract

**Highlights:**

**What are the main findings?**

- Weight-status distribution and specific dietary behaviours differed between two independent cross-sectional cohorts of Spanish primary schoolchildren assessed in 2018 and 2024, although the overall adjusted association with weight status was not statistically significant. Mean BMI and overall Mediterranean diet adherence did not differ significantly between cohorts.
- Children in the 2024 cohort reported lower social, emotional, and family self-concept scores after adjustment for age and sex.

**What are the implications of the main findings?**

- Monitoring Mediterranean diet adherence and specific dietary behaviours alongside weight status and self-concept may provide a more comprehensive assessment of child health.
- An integrated approach to children’s nutritional, physical, and psychosocial well-being may help inform comprehensive health-promotion strategies in school settings.

**Abstract:**

**Background/Objectives:** Comparing children’s dietary patterns, weight status, and self-concept across assessment periods may provide a broader perspective on child health. This study aimed to examine differences in Mediterranean diet adherence, weight status, and self-concept between two independent cross-sectional cohorts of primary schoolchildren assessed in 2018 and 2024. **Methods:** This comparative cross-sectional study included 419 primary schoolchildren attending a single school in Melilla, Spain, recruited through convenience sampling in 2018 (*n* = 188) and 2024 (*n* = 231). Body mass index (BMI) was used to classify weight status. Mediterranean diet adherence and self-concept were assessed using the Mediterranean Diet Quality Index for Children and Adolescents (KIDMED) and the Form 5 Self-Concept (AF5) Questionnaire, respectively. General linear and multinomial logistic regression models examined between-cohort differences adjusted for age and sex. **Results:** Mean BMI did not differ significantly between cohorts, and adjusted KIDMED scores did not differ significantly. Weight-status distributions differed before adjustment, but the overall adjusted association with assessment year was not statistically significant. Social, emotional, and family self-concept scores were lower in 2024 after adjustment (mean differences [2024 − 2018]: −0.493, −0.641, and −0.609, respectively; all *p* < 0.001), with similar sensitivity estimates. No KIDMED item survived Bonferroni correction. Exploratory analyses suggested school-grade interactions for some self-concept outcomes, but no statistically significant sex interactions. **Conclusions:** Children assessed in 2024 had lower social, emotional, and family self-concept scores after adjustment, despite no statistically significant differences in mean BMI or overall diet adherence. Adjusted evidence of weight-status differences was less consistent. These findings support considering psychosocial, anthropometric, and dietary indicators together when monitoring child health in similar school settings.

## 1. Introduction

The transition from childhood to adolescence is a critical period for developing and consolidating lifestyle habits that may influence health throughout the life course [1]. This period is also associated with an increased risk of overweight and obesity due to substantial metabolic, physical, and social changes [2]. Obesity is currently considered a major public health concern that seriously affects the health of children and adolescents [3,4]. Its development is influenced by genetic, environmental, psychosocial, and behavioural factors [5]. Low family-level socioeconomic status may also contribute to its development [6]. These factors are further compounded by the abandonment of healthy dietary patterns, such as the Mediterranean diet, whose nutritional, cultural, and socioeconomic benefits are widely recognized in the literature [7].

The Mediterranean diet is characterized by a high intake of plant-based foods, including fruit, seasonal vegetables, legumes, nuts, and olive oil, together with a lower intake of red meat [8,9,10]. It provides substantial amounts of plant-based proteins, low-glycaemic-index carbohydrates, monounsaturated fatty acids, dietary fibre, and antioxidants [11], which may contribute to cardiovascular health and reduce cardiovascular risk [12], while also supporting healthy body composition and reducing the risk of overweight and obesity [13]. Despite its benefits, a decline in adherence to this dietary pattern has been observed among schoolchildren in recent years [8,14], which may have implications for both current and future health [15]. Lower adherence to the Mediterranean diet may also be associated with less favourable adiposity outcomes in children and adolescents [16]. Excess weight, in turn, has been associated with differences in psychological characteristics such as self-esteem and self-concept [17,18], which are important components of children’s health and psychosocial well-being. Beyond its relationship with weight status, adherence to healthy dietary patterns may also be associated with children’s psychological functioning [19]. Dietary behaviours are also shaped by family and social contexts [20], which may be relevant to the development of children’s self-perceptions.

Self-concept is particularly relevant during childhood and adolescence given its influence on mental health, behaviour, and psychological well-being [21]. As a multidimensional construct, it encompasses physical, emotional, social, academic, and family domains [22], with global self-concept resulting from the integration of these domain-specific self-perceptions within a hierarchical structure [23,24]. Given its relevance to psychosocial development, self-concept may represent an important outcome to consider in school-based Physical Education (PE) and health-promotion initiatives [25], particularly because it has been associated with favourable psychosocial outcomes [26]. Therefore, examining self-concept alongside weight status and dietary patterns may provide a comprehensive perspective on the physical, behavioural, and psychosocial dimensions of health among primary school students.

Although previous studies have examined Mediterranean diet adherence and body mass index (BMI), including temporal changes in these variables, most have focused on adolescent populations or did not include self-concept. To our knowledge, evidence examining weight status, Mediterranean diet adherence, and self-concept simultaneously across comparable cohorts of primary schoolchildren remains limited. Accordingly, this study aimed to examine differences in weight status, Mediterranean diet adherence and self-concept between two independent cross-sectional cohorts of primary schoolchildren in grades 4–6 assessed in 2018 and 2024.

## 2. Materials and Methods

### 2.1. Study Design and Participants

A comparative cross-sectional design with two independent cohorts was used to examine weight status, adherence to the Mediterranean diet, and self-concept among primary schoolchildren. The study included 419 Spanish schoolchildren, an estimated 95% of whom were of Maghrebi origin. Of these, 188 were assessed in 2018 (*M_age_* = 10.61, *SD* = 0.92, 53.7% girls), and 231 in 2024 (*M_age_* = 10.90, *SD* = 1.04, 47.6% girls). The cohorts comprised different children, with no participants assessed at both time points. The 2024 cohort was slightly older on average than the 2018 cohort; age was included as a covariate in the multivariable analyses.

All participants attended the same primary school in Melilla, a Spanish autonomous city in North Africa. Both cohorts were drawn from the same school catchment area, and the eligibility criteria, grade levels included (grades 4–6), and recruitment procedures were substantially similar in 2018 and 2024. According to contextual information provided by the school, a substantial proportion of its pupils came from families experiencing socioeconomic disadvantage, including low household income and high unemployment. However, individual-level socioeconomic characteristics and other potentially relevant contextual factors were not systematically assessed in both cohorts, and their comparability between assessment years could therefore not be established.

Inclusion criteria were enrolment in fourth, fifth, or sixth grade of primary school, no diagnosed neurodevelopmental disorder, and complete questionnaire responses. The school was selected through convenience sampling because of its established collaboration with the Faculty of Education and Sport Sciences. The two samples consisted of independent groups of children, with no participants assessed in both 2018 and 2024.

### 2.2. Variables and Instruments

#### 2.2.1. Weight Status

BMI was calculated as weight (kg) divided by height squared (m^2^). Standing height was measured using a SECA 217 stadiometer (SECA GmbH, Hamburg, Germany) with a 1 mm graduation, and body weight was measured using a TANITA BC-543 bioelectrical impedance scale (Tanita Corporation, Tokyo, Japan). Weight status was classified according to the World Health Organization (WHO) 2007 BMI-for-age growth reference for school-aged children and adolescents, using age- and sex-specific thresholds [27]. For weight-status classification, age was grouped into six-month intervals (e.g., children aged 10 years and 2 months were assigned to the 10.0-year category, whereas those aged 10 years and 7 months were assigned to the 10.5-year category). Participants were classified as underweight (WHO thinness category; BMI-for-age < −2 *SD*), normal weight (−2 *SD* ≤ BMI-for-age ≤ +1 *SD*), overweight (+1 *SD* < BMI-for-age ≤ +2 *SD*), or obese (BMI-for-age > +2 *SD*). The same weighing scale and stadiometer were used in 2018 and 2024, and both instruments were calibrated in each assessment year.

#### 2.2.2. Mediterranean Diet

Adherence to the Mediterranean diet was assessed using the Mediterranean Diet Quality Index (KIDMED), originally developed by Serra-Majem et al. [28]. The questionnaire comprises 16 dichotomous (yes/no) items: 12 reflecting positive dietary behaviours and scored +1, whereas 4 reflect negative behaviours and are scored −1. The total score therefore ranges from −4 to 12, with higher scores indicating greater adherence. According to the original classification, participants were categorized as having low (≤3 points), medium (4–7 points), or high (≥8 points) adherence to the Mediterranean diet.

To calculate Cronbach’s alpha, the four negatively scored items were reverse-coded so that all items were oriented in the same direction, with higher values indicating healthier dietary behaviours. Cronbach’s alpha was 0.720 in the pooled sample and 0.561 and 0.620 in the 2018 and 2024 cohorts, respectively. The cohort-specific coefficients indicated limited internal consistency. These values should be interpreted in the context of the diverse dietary behaviours assessed by KIDMED, although they remain a limitation when interpreting between-cohort comparisons.

#### 2.2.3. Self-Concept

Self-concept was assessed using the Form 5 Self-Concept Questionnaire (AF5), developed by García and Musitu [29]. The AF5 is a validated 30-item instrument applicable across a broad age range, from childhood to adulthood. It comprises five dimensions: academic, social, emotional, family, and physical self-concept. Each item is rated on a 5-point Likert-type scale ranging from 1 (never) to 5 (always). Dimension scores were calculated according to the authors’ recommendations. The instrument has demonstrated acceptable psychometric properties, with an internal consistency coefficient (Cronbach’s α) of 0.760 reported in the original study. In the present study, Cronbach’s alpha for the full questionnaire in the overall sample was 0.755. Internal consistency was also examined separately for each dimension within each cohort. In the 2018 cohort, Cronbach’s alpha coefficients were 0.641 for academic self-concept, 0.552 for social self-concept, 0.463 for emotional self-concept, 0.610 for family self-concept, and 0.574 for physical self-concept. The corresponding coefficients in 2024 were 0.726, 0.448, 0.692, 0.539, and 0.647, respectively. These values indicated limited internal consistency for several dimensions, particularly emotional self-concept in 2018 and social self-concept in 2024. Findings involving dimensions with low internal consistency should therefore be interpreted with caution, particularly with regard to between-cohort comparisons. Formal measurement-invariance testing between cohorts was not performed.

### 2.3. Procedure

The same procedure was followed in 2018 and 2024. Authorization to conduct the study was obtained from the Provincial Directorate of the Ministry of Education, and permission was subsequently obtained from the participating school’s management team. The researchers held an information session with physical education (PE) teachers and school management to explain the study objectives and procedures. Written informed consent was obtained from the parents or legal guardians of all participants. In 2018, 271 information letters were distributed, and parental or guardian consent was obtained for 204 students; 16 were excluded because they were absent on the assessment day or submitted invalid questionnaires, resulting in a final sample of 188 participants. In 2024, 275 letters were distributed, and consent was obtained for 242 students; 11 were excluded for the same reasons, resulting in a final sample of 231 participants. Questionnaires were considered invalid if they contained unanswered items or showed a uniform response pattern across all items.

Data collection was conducted in October 2018 and October 2024, during the first term of the respective school years, using standardized procedures during regularly scheduled PE classes. After receiving detailed instructions from the researchers, PE teachers obtained the anthropometric measurements and administered the questionnaires. The same versions of the AF-5 and KIDMED questionnaires were used in both cohorts. Participant instructions, scoring procedures, and reverse-coding procedures were the same in both assessment years. Assessments were conducted by the same personnel, in the same educational settings, and at comparable early-morning times to support participants’ attention and concentration. The researchers provided all necessary materials and were present during the assessment sessions to provide support when needed. Anthropometric measurements were obtained using the same equipment in both assessment years, with participants barefoot and wearing light sportswear in an area used for PE activities under suitable environmental conditions. Questionnaires were administered in a regular classroom during scheduled PE classes. Confidentiality was maintained throughout the study, and participants were informed that their data would be used exclusively for scientific purposes.

### 2.4. Data Analysis

Statistical analyses were performed using IBM SPSS Statistics version 30.0 (IBM Corp., Armonk, NY, USA), with statistical significance set at *p* < 0.05 unless otherwise specified. Continuous variables were summarised as mean ± standard deviation (*SD*), and categorical variables as frequencies and percentages. Internal consistency was assessed using Cronbach’s alpha for the KIDMED items, the full AF5 questionnaire, and each AF5 dimension.

Unadjusted comparisons between cohorts used independent-samples *t*-tests, with Welch’s correction when Levene’s test indicated unequal variances. Unadjusted mean differences (2024 minus 2018) and 95% confidence intervals (CIs) were also reported for BMI and KIDMED scores. Effect sizes were expressed as absolute values of Cohen’s *d*. Categorical variables were compared using Pearson’s chi-square test, with Cramér’s *V* as the effect-size measure. The Fisher–Freeman–Halton exact test was used to compare weight-status distributions because of sparse cell counts. Values of 0.20, 0.50, and 0.80 for Cohen’s *d* and 0.10, 0.30, and 0.50 for Cramér’s *V* were interpreted as small, moderate, and large effects, respectively.

Adjusted between-cohort differences in self-concept and KIDMED scores were estimated using general linear models, with assessment year as the main explanatory variable and age and sex as covariates. Results were reported as adjusted mean differences (2024 minus 2018) with 95% confidence intervals (CIs). An exploratory KIDMED model was additionally adjusted for BMI and overall self-concept. Age was included to account for developmental differences within school grades. Because age and grade were strongly correlated (Spearman’s ρ = 0.778), they were not entered together; sensitivity analyses replaced age with grade while retaining sex.

Weight status was analysed using multinomial logistic regression, with normal weight as the reference category. Children classified as underweight were excluded from these models because their small number prevented stable estimation. Models were adjusted for age and sex, with grade replacing age in sensitivity analyses. Adjusted odds ratios (ORs), 95% CIs, and likelihood-ratio tests for the overall association with assessment year were reported.

Exploratory two-way analyses of variance examined whether between-cohort differences in BMI, KIDMED score, overall self-concept, and its five dimensions varied by sex or school grade. Models included assessment year, sex or grade, and their interaction. Effect sizes were expressed as partial eta squared (η^2^p). Significant year-by-grade interactions were followed by Bonferroni-adjusted pairwise comparisons.

Supplementary binary logistic regression was used to identify variables independently associated with membership in the 2024 cohort (2018 = 0; 2024 = 1). Model 1 included age, sex, BMI, KIDMED score, and overall self-concept; Model 2 replaced overall self-concept with its five dimensions. These exploratory models did not estimate the primary adjusted between-cohort differences. Regression coefficients, ORs, 95% CIs, and *p*-values were reported. Model fit was assessed using the omnibus and Hosmer–Lemeshow tests and Cox–Snell and Nagelkerke pseudo-*R*^2^ statistics. Multicollinearity was assessed using variance inflation factors (VIFs).

Exploratory comparisons of individual KIDMED items were performed using Pearson’s chi-square tests. These analyses were not prespecified. Differences in the percentage of affirmative responses (2024 minus 2018) were reported in percentage points, with unadjusted 95% CIs calculated using Newcombe’s method. Bonferroni correction was applied across the 16 comparisons (*p* < 0.003125).

## 3. Results

### 3.1. Demographic, Anthropometric, and Dietary Characteristics by Assessment Year

Table 1 summarizes demographic characteristics, anthropometric variables, weight status, and Mediterranean diet adherence of children assessed in 2018 and 2024. Children in the 2024 cohort were slightly older than those in the 2018 cohort (10.90 ± 1.04 vs. 10.61 ± 0.92 years; *p* = 0.003), although the difference was small. The global comparison of weight-status distribution yielded an unadjusted *p*-value of 0.024 (two-sided Fisher–Freeman–Halton exact test, *p* = 0.024; Cramér’s *V* = 0.15). Descriptively, the proportion of children classified as underweight decreased from 3.7% in 2018 to 0.4% in 2024. Compared with the 2018 cohort, the 2024 cohort had lower proportions of children classified as underweight and overweight and higher proportions classified as normal-weight and obese. No statistically significant between-cohort differences were observed in sex, school grade, body weight, height, BMI, KIDMED score, or Mediterranean diet adherence (all *p* > 0.05). The unadjusted between-cohort differences (2024 minus 2018) were 0.01 kg/m^2^ for BMI (95% CI: −0.65 to 0.67) and 0.23 points for the KIDMED score (95% CI: −0.23 to 0.69). These results indicate that no statistically significant differences were detected, rather than establishing equivalence or stability between cohorts.

### 3.2. Cohort Differences in Overall and Multidimensional Self-Concept

Unadjusted self-concept scores are presented in Table 2. Overall self-concept was lower in the 2024 cohort than in the 2018 cohort (3.51 ± 0.32 vs. 3.86 ± 0.47; *p* < 0.001; Cohen’s *d* = 0.881). Social, emotional, and family self-concept scores were also significantly lower in the 2024 cohort (all *p* < 0.001), with Cohen’s *d* values of 0.814, 0.711, and 1.054, respectively. No statistically significant unadjusted differences were found in academic self-concept (*p* = 0.667; *d* = 0.042) or physical self-concept (*p* = 0.067; *d* = 0.181).

Adjusted between-cohort differences were estimated using general linear models with assessment year as the exposure indicator. Model 1 adjusted for age and sex. Because age and school grade were strongly correlated (Spearman’s *ρ* = 0.778, *p* < 0.001), they were not included simultaneously; Model 2, adjusted for school grade and sex, was therefore fitted as a sensitivity analysis. In Model 1, children assessed in 2024 had lower adjusted mean scores than those assessed in 2018 for overall self-concept (adjusted mean difference, 2024 minus 2018: −0.350; 95% CI: −0.428 to −0.272; *p* < 0.001), as well as for social, emotional, and family self-concept (Table 3). Adjusted estimates for academic self-concept did not differ between cohorts. The estimate for physical self-concept was negative but did not reach statistical significance in Model 1 (−0.118; 95% CI: −0.252 to 0.017; *p* = 0.086).

Estimates were similar in the school-grade– and sex-adjusted sensitivity model. However, the difference in physical self-concept reached statistical significance only in this model (adjusted mean difference: −0.134; 95% CI: −0.265 to −0.002; *p* = 0.046). This finding was not adjusted for multiple testing and should be interpreted as exploratory rather than confirmatory.

### 3.3. Interaction Effects of Sex and School Grade on Cohort Differences

Exploratory interaction analyses examined whether between-cohort differences varied by sex or school grade. No significant year × sex interactions were observed for BMI, KIDMED score, overall self-concept, or any self-concept dimension (all interaction *p* > 0.05).

Nominally significant year × grade interactions were observed for overall, academic, emotional, family, and physical self-concept, but not for BMI, KIDMED score, or social self-concept. Bonferroni-adjusted comparisons showed that overall self-concept was lower in 2024 across all grades; emotional and family self-concept were lower in fifth and sixth grade; and physical self-concept was lower in fifth grade only. For academic self-concept, none of the within-grade between-cohort comparisons reached statistical significance after Bonferroni adjustment, despite the nominally significant interaction.

Detailed interaction statistics, grade-specific estimated marginal means, and Bonferroni-adjusted between-cohort contrasts are provided in Supplementary Table S1. Given their exploratory nature and the number of outcomes examined, these findings should be interpreted cautiously.

### 3.4. Adjusted Between-Cohort Differences in Dietary Adherence and Weight Status

Adjusted KIDMED scores did not differ significantly between cohorts in the age- and sex-adjusted model (adjusted mean difference, 2024 minus 2018: 0.177; 95% CI: −0.285 to 0.640; *p* = 0.451). The school-grade– and sex-adjusted sensitivity model yielded a similar estimate (0.243; 95% CI: −0.209 to 0.695; *p* = 0.292).

In the age- and sex-adjusted multinomial model, the association between cohort and overweight relative to normal weight was nominally significant (adjusted OR = 0.619; 95% CI: 0.391–0.979; *p* = 0.040). No statistically significant association was observed for obesity relative to normal weight (adjusted OR = 1.027; 95% CI: 0.574–1.835; *p* = 0.930). However, the overall association between assessment year and weight status was not statistically significant (likelihood-ratio test, *p* = 0.098). The overweight estimate was closer to the null in the school-grade– and sex-adjusted sensitivity model, and its confidence interval included 1 (adjusted OR = 0.666; 95% CI: 0.423–1.049; *p* = 0.080). Taken together, these findings did not provide robust evidence of adjusted between-cohort differences in weight status. Results are summarised in Table 4.

### 3.5. Complementary Analyses of Variables Associated with Cohort Membership

An exploratory general linear model was fitted to compare KIDMED scores between cohorts, adjusting for age, sex, BMI, and overall self-concept. In this model, scores were higher in the 2024 cohort than in the 2018 cohort (adjusted mean difference, 2024 minus 2018: 0.595 points; 95% CI: 0.100–1.090; *p* = 0.019). This association was not observed in the unadjusted or age- and sex-adjusted analyses, suggesting that the finding depended on the covariates included.

A complementary multivariable binary logistic regression model examined characteristics associated with membership in the 2024 cohort. The overall model was statistically significant (Omnibus test: χ^2^(5) = 88.81, *p* < 0.001) with Nagelkerke and Cox–Snell pseudo-*R*^2^ values of 0.256 and 0.191, respectively. Results are presented in Table 5.

Older age, higher KIDMED scores, and lower overall self-concept showed nominally significant associations with membership in the 2024 cohort after mutual covariate adjustment, whereas sex and BMI were not (Table 5). For each one-unit increase in overall self-concept score, the adjusted odds of belonging to the 2024 cohort were multiplied by 0.093, holding the other variables in the model constant.

A second multivariable binary logistic regression model was fitted, replacing overall self-concept with its five dimensions. The model was statistically significant (Omnibus test: χ^2^(9) = 162.506, *p* < 0.001), with a Nagelkerke *R*^2^ of 0.430 and Cox & Snell *R*^2^ of 0.321. The results are presented in Table 6.

With all five dimensions included simultaneously and adjustment for age, sex, BMI, and KIDMED score, higher social, emotional, and family self-concept scores were associated with lower odds of membership in the 2024 cohort (all *p* < 0.001). Academic and physical self-concept were not statistically significantly associated with cohort membership. Older age and higher KIDMED scores remained associated with membership in the 2024 cohort, whereas sex and BMI did not.

These exploratory findings should not be interpreted as evidence of an overall improvement in Mediterranean diet adherence. No statistically significant differences in KIDMED scores were detected in the unadjusted or age- and sex-adjusted comparisons (Table 1 and Table 4), and the association observed in the exploratory models depended on the covariates included. The nominally significant KIDMED associations were not adjusted for multiple testing and require confirmation in independent samples.

### 3.6. Between-Cohort Comparisons of Individual KIDMED Items

Although no statistically significant unadjusted differences were observed in the overall KIDMED score, six individual items showed nominally significant between-cohort differences before correction for multiple comparisons (Table 7). Descriptively, children assessed in 2024 were less likely to report visiting fast-food restaurants more than once per week, consuming breakfast cereals, or consuming two yoghurts and/or 40 g of cheese daily. Conversely, higher proportions reported regular nut consumption, using olive oil at home, and having a dairy product for breakfast. However, none of these item-level differences remained statistically significant after Bonferroni correction for the 16 comparisons (adjusted significance threshold: *p* < 0.003125). These analyses were exploratory and not prespecified, and their findings should therefore be interpreted cautiously. KIDMED item scores reflect the index’s predefined scoring rules rather than comprehensive assessments of the nutritional quality of individual foods.

### 3.7. Overview of Cohort Differences Across Study Outcomes

**Figure 1:**
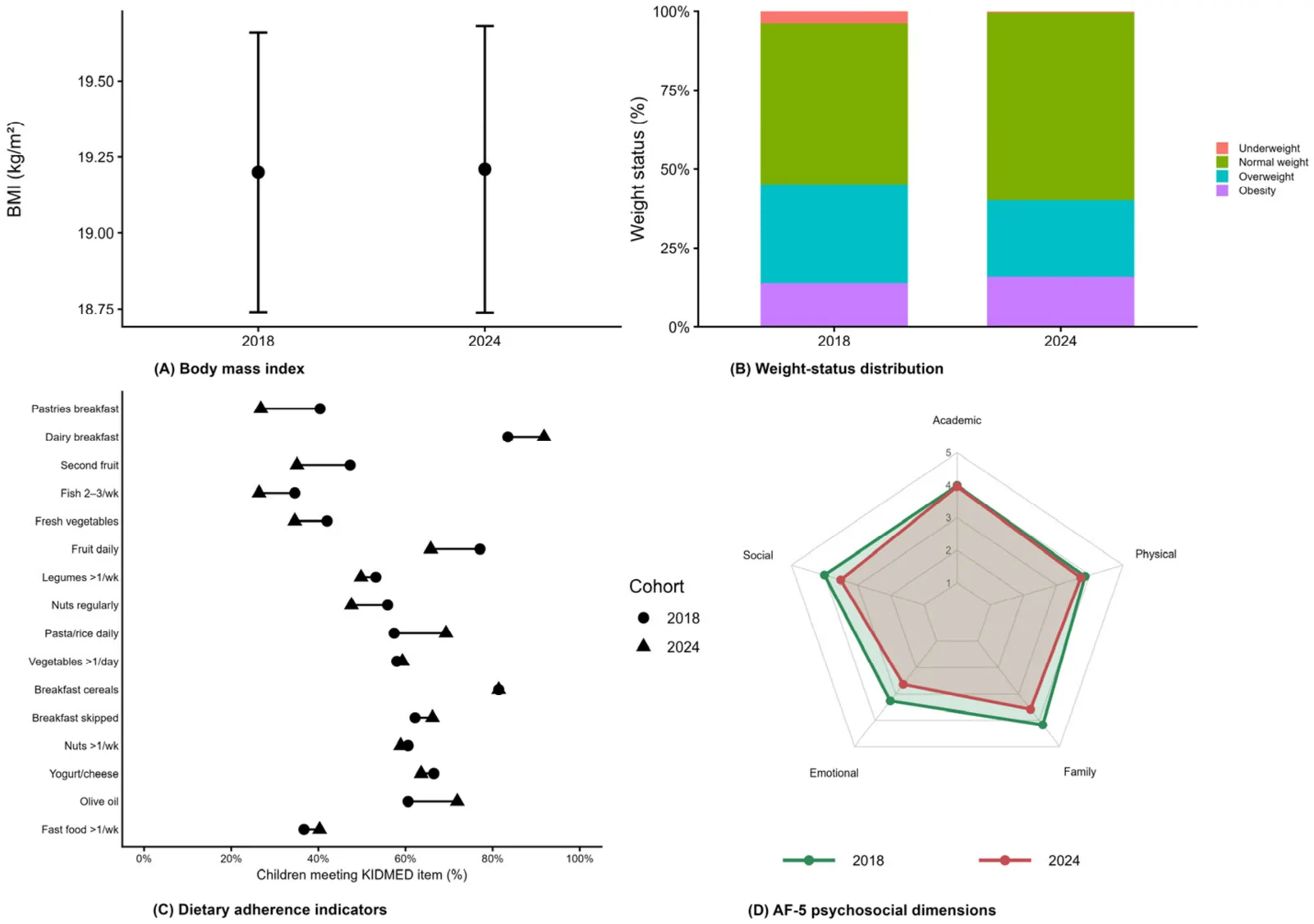
Overview of anthropometric, weight-status, dietary, and self-concept outcomes between the 2018 and 2024 cohorts. BMI is presented as mean ± 95% confidence interval. The stacked bars show weight-status distributions; the point-range plot shows the percentage of children reporting an affirmative response to each KIDMED item; and the radar chart presents mean self-concept scores by dimensions. KIDMED item-level comparisons were exploratory; none remained statistically significant after Bonferroni correction for 16 comparisons (significance threshold: *p* < 0.003125).

Figure 1 summarises the findings across anthropometric, weight-status, dietary, and self-concept outcomes. No statistically significant unadjusted differences were detected in mean BMI or KIDMED scores. Weight-status distributions differed in the unadjusted analysis, although adjusted findings were not robust. Overall, social, emotional, and family self-concept scores were lower in the 2024 cohort in both unadjusted and adjusted analyses. No individual KIDMED item remained statistically significant after Bonferroni correction.

## 4. Discussion

This study examined differences in Mediterranean diet adherence, weight status, and self-concept between two independent cohorts of fourth- to sixth-grade primary school students assessed in 2018 and 2024. Overall, the cohorts were broadly comparable in sex distribution, school grade, anthropometric measures, KIDMED scores, and Mediterranean diet adherence. Children assessed in 2024 were slightly older, although the difference was small (*d* = 0.36), suggesting limited practical relevance despite statistical significance. Nevertheless, age was included as a covariate because of its potential association with psychosocial and weight-related outcomes.

The unadjusted comparison of weight-status distributions was nominally significant (*p* = 0.024), with a small association magnitude (Cramér’s *V* = 0.15). This finding was not adjusted for multiple testing and should be interpreted as exploratory rather than confirmatory. Descriptively, the 2024 cohort had a higher proportion of children with normal weight and lower proportions classified as underweight or overweight, while the proportion classified as obese was slightly higher in 2024 (16.0% vs. 13.8%). However, mean BMI did not differ significantly between cohorts, indicating that the difference in weight-status distribution was not accompanied by a statistically significant difference in mean BMI. Evidence of adjusted between-cohort differences in weight status was not robust.

Excess weight was highly prevalent in both cohorts, affecting 45.2% of participants in 2018 and 40.2% in 2024. These estimates are broadly consistent with those reported by Homs et al. [9], who found approximately 24% of primary schoolchildren to be overweight and 15% to be obese. Although they reported a longitudinal increase in overweight prevalence, the lower proportion of overweight observed in our 2024 cohort was descriptive and should not be interpreted as evidence of a temporal decline. Conversely, Pinel Martínez et al. [2] reported substantially lower prevalence estimates for overweight (3.8%) and obesity (5.4%). Such differences should be interpreted cautiously, as estimates of childhood overweight and obesity may vary according to the age and characteristics of the study population, geographical setting, assessment period [30], and criteria used to define weight status [31]. Nevertheless, the relatively high prevalence of excess weight observed in both cohorts highlights the persistence of overweight and obesity as relevant health concerns among school-aged children in this setting. From a public-health perspective, these findings highlight the importance of evaluating whether preventive actions promoted by educational and health authorities achieve sustained reductions in childhood overweight and obesity. However, this interpretation should be made cautiously, as the present study did not assess the implementation or effectiveness of specific intervention programmes.

Regarding adherence to the Mediterranean diet, no significant unadjusted differences were observed between cohorts, either in continuous KIDMED scores or across low, medium, and high adherence levels. Low adherence was slightly less common in 2024 than in 2018 (11.7% vs. 13.8%), whereas high adherence was slightly more common (32.0% vs. 30.3%). However, the overall distribution of adherence levels did not differ significantly between cohorts. Medium adherence remained the most common category in both cohorts (55.9% in 2018 and 56.3% in 2024). Approximately one-third of children showed high adherence, whereas low adherence was less common. These findings indicate that although most children showed medium or high adherence to the Mediterranean diet, medium adherence remained predominant, leaving room for further improvement in this school-aged population. This pattern is broadly consistent with previous studies of Spanish children and adolescents, which have generally reported suboptimal or intermediate rather than optimal adherence [9,32,33,34,35]. However, comparisons should be interpreted cautiously because of differences in age, geographical context, sample composition, assessment period, and classification procedures [8].

Although overall adherence did not differ significantly, six individual KIDMED items showed nominally significant differences before correction for multiple testing. However, none remained statistically significant after Bonferroni correction for the 16 comparisons. According to the KIDMED scoring criteria, the 2024 cohort showed more favourable patterns for visits to fast-food restaurants, nut consumption, olive-oil use at home, and consumption of a dairy product at breakfast, but less favourable patterns for breakfast cereal consumption and daily consumption of two yoghurts or 40 g of cheese. Several other items showed numerically lower reported consumption in 2024, including a second daily fruit, vegetables more than once daily, fish, and pasta or rice, although these differences were not statistically significant; commercially baked goods or pastries were numerically more common, but this difference was also non-significant. Thus, the item-level results showed a mixed descriptive pattern rather than a uniform change in dietary habits.

This pattern differs from the findings of Herrera-Ramos et al., who reported an overall worsening of Mediterranean diet adherence among Spanish children and adolescents when comparing the enKid (1998–2000) and PASOS (2019–2020) studies [8]. However, their reported increases in olive oil and nut consumption were directionally consistent with the descriptive differences in these items in the 2024 cohort, although neither between-cohort comparison remained statistically significant after Bonferroni correction. In contrast, our findings showed a descriptively lower proportion reporting visits to fast-food restaurants, while no statistically significant overall difference in KIDMED scores was detected. Differences between studies may reflect variation in assessment periods, population characteristics, and geographical settings.

The item-level results suggest possible variation in the direction of dietary behaviours, but none of the comparisons remained statistically significant after multiplicity correction. They therefore do not establish differences in specific dietary behaviours or explain the absence of a statistically significant difference in the overall KIDMED score. These descriptive observations may inform hypotheses for future research but should not be interpreted as evidence of dietary change or longitudinal decline. Future studies should examine individual dietary behaviours alongside contextual factors, such as household income, parental education, the local availability of fast-food outlets, and energy-drink consumption, to clarify the factors associated with changes in children’s dietary patterns.

In terms of self-concept, both overall self-concept and several dimensions showed significantly lower scores in the 2024 cohort than in the 2018 cohort. Overall self-concept showed a large effect size (Cohen’s *d* = 0.88), as did social and family self-concept (*d* = 0.81 and 1.05, respectively), whereas the effect size for emotional self-concept was moderate (*d* = 0.71). Scores were higher in 2018, with the largest difference observed in family self-concept. No significant unadjusted differences were found in academic (*p* = 0.667) or physical self-concept (*p* = 0.067), although both estimates indicated lower scores in 2024. Thus, the between-cohort differences followed a domain-specific pattern, primarily involving social, emotional, and family self-concept. The difference in physical self-concept was nominally significant only in the sensitivity model adjusted for school grade and sex without correction for multiple testing, and should therefore be interpreted as exploratory rather than confirmatory.

The exploratory interaction analyses suggested that some between-cohort differences in self-concept might vary by school grade, although interaction *p*-values were not adjusted for multiple testing across outcomes. Bonferroni-adjusted within-grade between-cohort comparisons showed lower emotional and family self-concept in fifth and sixth grades and lower physical self-concept in fifth grade only. Although the year × grade interaction for academic self-concept was nominally significant, no individual within-grade between-cohort comparison reached statistical significance after Bonferroni adjustment. No significant year × sex interactions were observed for BMI, KIDMED score, overall self-concept, or any self-concept dimension, suggesting that the cohort-related differences did not vary detectably by sex. These findings should be interpreted cautiously because interaction and subgroup analyses may have limited statistical power and should be considered hypothesis-generating rather than definitive evidence of effect modification.

Evidence on differences in multidimensional self-concept between assessment periods among primary schoolchildren is limited. A previous study using the AF-5 in primary schoolchildren reported lower scores in emotional self-concept and relatively higher scores in family self-concept [36]. Although emotional self-concept also showed a moderate between-cohort difference in our study, family self-concept had the largest effect size, followed by social and emotional self-concept. However, low internal consistency in emotional self-concept in 2018 and social self-concept in 2024 introduces uncertainty into the interpretation of the corresponding between-cohort differences and their estimated magnitudes. Furthermore, measurement invariance between cohorts has not been established, so score differences cannot be assumed to reflect differences in the underlying constructs alone. The observed domain-specific pattern should therefore be interpreted cautiously rather than attributed to particular developmental, social, or family-related influences.

Given that the present study was not designed to identify causal mechanisms, the observed differences between the 2018 and 2024 cohorts may reflect unmeasured cohort-specific characteristics, potential measurement-related factors, and broader social and environmental circumstances. In this context, children’s engagement with digital environments may be relevant to understanding self-concept development. Social media use may be associated with children’s and young adolescents’ self-evaluations and emotional well-being, with upward social comparison representing one potential explanatory mechanism [37]. Similarly, engagement with digital activities may influence the amount and nature of the time that children spend in family activities and interactions, potentially shaping their perceptions of family relationships [38]. However, these interpretations should be treated cautiously because social media use, technology exposure, and family interaction patterns were not assessed in the present study.

As a supplementary exploratory approach, the logistic regression models examined the joint associations of the study variables with cohort membership, complementing the primary analyses of adjusted between-cohort differences. In the first model, which included sex, BMI, age, KIDMED score, and overall self-concept, older age, higher KIDMED scores, and lower overall self-concept were associated with higher odds of membership in the 2024 cohort after mutual adjustment, whereas sex and BMI were not. The association between lower overall self-concept and membership in the 2024 cohort was consistent with the lower scores observed in the between-cohort comparisons and indicated that this association persisted after accounting for the other variables included in the model.

The association with higher KIDMED scores was nominally significant and was not adjusted for multiple testing. They should therefore be interpreted as exploratory rather than confirmatory, particularly because overall KIDMED scores did not differ significantly between cohorts in the unadjusted or age- and sex-adjusted analyses. The difference between the unadjusted and adjusted findings may reflect the influence of covariate adjustment, although the present analyses do not establish which relationships among the included variables account for this pattern. In the second model, which included the individual self-concept dimensions in place of overall self-concept, social, emotional, and family self-concept were associated with membership in the 2024 cohort, whereas academic and physical self-concept were not. This pattern broadly aligned with the unadjusted comparisons, with associations involving the social, emotional, and family domains. Overall, the models identified exploratory, covariate-adjusted associations; their *p*-values were not corrected for multiplicity, and they did not establish causal relationships or clarify the mechanisms underlying the differences between cohorts. Because the study used two independent cross-sectional cohorts, the odds ratios should be interpreted as conditional measures of association rather than as direct estimates of risk or probability.

This study has several limitations. First, its cross-sectional design and the use of two independent cohorts preclude assessment of individual changes over time and limit causal interpretation. Although participants were recruited from the same school and the two cohorts had broadly comparable characteristics, unmeasured cohort-specific factors and differential non-response may have influenced the findings. Recruitment from a single school in a Spanish autonomous city in North Africa also limits generalisability. Differences in participation rates between the two cohorts may reflect differential non-response and could have introduced selection bias.

Second, individual-level data on household income, parental education, family structure, migration generation, and other socioeconomic indicators were unavailable, preventing systematic comparisons and adjustment for these factors. School-provided socioeconomic information should therefore be regarded as contextual rather than as individually measured exposures. Digital media use and family interaction patterns were also not assessed; consequently, the cohort differences cannot be attributed to these factors or specifically to the COVID-19 pandemic.

Third, the use of self-reported dietary and self-concept data may have introduced recall or social-desirability bias, particularly in the assessment of dietary behaviours and self-concept. Although the same AF-5 version, language, administration procedures, and scoring conventions were used in both cohorts, internal consistency varied across dimensions and assessment years. In particular, emotional self-concept in 2018 (Cronbach’s α = 0.463) and social self-concept in 2024 (α = 0.448) showed low internal consistency, introducing uncertainty into the interpretation of the corresponding between-cohort differences and their estimated magnitudes. These findings therefore warrant particular caution. Identical questionnaire versions and administration procedures do not, by themselves, establish measurement equivalence between cohorts; consequently, some of the observed between-cohort differences may partly reflect differences in measurement properties rather than differences in the underlying self-concept constructs. Differences in classroom dynamics, language-related factors, cultural context, or item interpretation may also have influenced responses. Formal measurement-invariance testing and item-level response-pattern analyses were not performed; therefore, the observed score differences cannot be assumed to reflect differences in the underlying self-concept constructs alone.

Finally, although Bonferroni correction was applied to individual KIDMED item comparisons and post-hoc grade comparisons, no study-wide adjustment for multiple testing was performed across all outcomes and exploratory analyses. Findings based on nominal *p*-values, particularly those close to 0.05, should therefore be interpreted as exploratory rather than confirmatory. In addition, the supplementary logistic regression models examining cohort membership were descriptive and exploratory rather than clinically predictive or causal, which limits their epidemiological interpretation.

Future studies should use longitudinal designs with repeated assessments and include larger samples from multiple schools and geographical settings, incorporating individual socioeconomic indicators, digital media use, family interactions, and other psychosocial factors. Examining both overall Mediterranean diet adherence and individual dietary behaviours would also provide a more detailed account of dietary patterns.

Despite these limitations, the findings support considering physical, dietary, and psychosocial dimensions together when monitoring child health. The descriptive differences in weight-status distribution and lower self-concept scores in the 2024 cohort, alongside no statistically significant differences in mean BMI or overall Mediterranean diet adherence, illustrate how these indicators provided different perspectives on the study population. Whether multidimensional monitoring improves school- or paediatric-based health-promotion strategies requires further evaluation.

## 5. Conclusions

The comparison of two independent cross-sectional cohorts of primary schoolchildren assessed in 2018 and 2024 identified lower self-concept scores in the 2024 cohort, particularly in the social, emotional, and family domains, whereas mean BMI and overall Mediterranean diet adherence did not differ significantly between cohorts. The interpretation of the self-concept differences is limited by low internal consistency in some dimensions and the absence of measurement-invariance evidence. Differences in weight-status distribution were nominally significant in the unadjusted analysis, but adjusted findings were not robust. The 2024 cohort had a numerically higher prevalence of obesity alongside lower proportions of underweight and overweight. Individual KIDMED items showed mixed descriptive patterns, but none of the between-cohort comparisons remained statistically significant after Bonferroni correction. Given the single-school convenience sample and its specific sociocultural characteristics, these findings should not be generalized to Spanish primary schoolchildren as a whole. Nevertheless, this study highlights the potential value of considering physical, dietary, and psychosocial indicators together when monitoring child health in school settings.

## Figures and Tables

**Table 1:** Characteristics of the study sample by assessment year.

Variable	2018 (*n* = 188)	2024 (*n* = 231)	Total (*n* = 419)	*p*-Value	Cramér’s *V*
Age (years)	10.61 ± 0.92	10.90 ± 1.04	10.77 ± 1.00	0.003 *	—
Sex				0.214	0.061
Boys	87 (46.3%)	121 (52.4%)	208 (49.6%)		
Girls	101 (53.7%)	110 (47.6%)	211 (50.4%)		
School grade				0.516	0.056
Grade 4	57 (30.3%)	79 (34.2%)	136 (32.5%)		
Grade 5	62 (33.0%)	79 (34.2%)	141 (33.7%)		
Grade 6	69 (36.7%)	73 (31.6%)	142 (33.9%)		
Weight (kg)	40.48 ± 8.19	40.82 ± 10.37	40.67 ± 9.45	0.710	—
Height (cm)	145.12 ± 10.97	144.99 ± 8.28	145.05 ± 9.57	0.893	—
BMI (Kg/m^2^)	19.20 ± 3.22	19.21 ± 3.66	19.20 ± 3.46	0.974	—
KIDMED score	6.10 ± 2.46	6.33 ± 2.28	6.22 ± 2.36	0.319	—
Weight status				0.024 *	0.150
Underweight	7 (3.7%)	1 (0.4%)	8 (1.9%)		
Normal weight	96 (51.1%)	137 (59.3%)	233 (55.6%)		
Overweight	59 (31.4%)	56 (24.2%)	115 (27.4%)		
Obesity	26 (13.8%)	37 (16.0%)	63 (15.0%)		
Mediterranean diet adherence				0.788	0.034
Low adherence	26 (13.8%)	27 (11.7%)	53 (12.6%)		
Medium adherence	105 (55.9%)	130 (56.3%)	235 (56.1%)		
High adherence	57 (30.3%)	74 (32.0%)	131 (31.3%)		

Legend: BMI, body mass index; KIDMED, Mediterranean Diet Quality Index for Children and Adolescents. *SD*, standard deviation. Values are presented as mean ± *SD* or *n* (%). Continuous variables were compared using independent-samples *t*-tests or Welch’s *t*-tests, as appropriate. Categorical variables were compared using Pearson’s chi-square tests, except for weight status, for which the Fisher–Freeman–Halton exact test was used because of sparse cell counts. Cramér’s *V* quantifies the magnitude of categorical associations. All *p*-values are unadjusted for multiple testing. An asterisk (*) indicates nominal statistical significance (*p* < 0.05), which should not be interpreted as confirmatory evidence.

**Table 2:** Comparison of overall self-concept and its dimensions between the 2018 and 2024 cohorts.

Variable	2018 (*n* = 188)	2024 (*n* = 231)	*p*-Value	Cohen’s *d*
Overall self-concept	3.86 ± 0.47	3.51 ± 0.32	<0.001 *	0.881
Academic	3.99 ± 0.69	3.96 ± 0.66	0.667	0.042
Social	4.00 ± 0.70	3.51 ± 0.50	<0.001 *	0.814
Emotional	3.26 ± 0.95	2.63 ± 0.84	<0.001 *	0.711
Family	4.18 ± 0.73	3.58 ± 0.39	<0.001 *	1.054
Physical	3.86 ± 0.70	3.73 ± 0.68	0.067	0.181

Legend: Values are presented as mean ± standard deviation (*SD*). Comparisons between the 2018 and 2024 cohorts were performed using the independent-samples *t*-test or Welch *t*-test, as appropriate, for continuous variables. Absolute Cohen’s *d* indicates the magnitude of between-cohort differences. An asterisk (*) indicates nominal statistical significance (*p* < 0.05); *p*-values were not adjusted for multiple testing.

**Table 3:** Adjusted between-cohort differences in overall self-concept and its dimensions.

Self-Concept Outcome	Model 1: Adjusted Difference (95% CI)	*p*-Value	Model 2: Adjusted Difference (95% CI)	*p*-Value
Overall self-concept	−0.350 (−0.428 to −0.272)	<0.001	−0.341 (−0.417 to −0.264)	<0.001
Academic self-concept	−0.018 (−0.150 to 0.114)	0.787	−0.015 (−0.145 to 0.115)	0.821
Social self-concept	−0.493 (−0.612 to −0.375)	<0.001	−0.484 (−0.602 to −0.366)	<0.001
Emotional self-concept	−0.641 (−0.814 to −0.467)	<0.001	−0.610 (−0.781 to −0.439)	<0.001
Family self-concept	−0.609 (−0.721 to −0.497)	<0.001	−0.585 (−0.693 to −0.478)	<0.001
Physical self-concept	−0.118 (−0.252 to 0.017)	0.086	−0.134 (−0.265 to −0.002)	0.046

Legend: Values are adjusted mean differences, calculated as 2024 minus 2018; negative values indicate lower scores in the 2024 cohort. Model 1 was adjusted for age and sex. Model 2 was adjusted for school grade and sex and was specified as a sensitivity analysis because age and school grade were strongly correlated (Spearman’s *ρ* = 0.778, *p* < 0.001). Adjustment refers to covariate adjustment, not correction for multiple testing. All *p*-values are unadjusted for multiplicity, and nominally significant findings should not be interpreted as confirmatory evidence.

**Table 4:** Adjusted between-cohort differences in KIDMED score and weight-status categories.

Outcome	Model 1: Adjusted Estimate (95% CI)	*p*-Value	Model 2: Adjusted Estimate (95% CI)	*p*-Value
KIDMED score, adjusted mean difference (95% CI)	0.177 (−0.285 to 0.640)	0.451	0.243 (−0.209 to 0.695)	0.292
Overweight vs. normal weight, adjusted OR (95% CI)	0.619 (0.391–0.979)	0.040	0.666 (0.423–1.049)	0.080
Obesity vs. normal weight, adjusted OR (95% CI)	1.027 (0.574–1.835)	0.930	0.935 (0.525–1.664)	0.818

Legend: KIDMED, Mediterranean Diet Quality Index for Children and Adolescents; CI, confidence interval; OR, odds ratio. Estimates are adjusted mean differences (2024 minus 2018) for KIDMED and adjusted ORs comparing 2024 with 2018 for overweight or obesity relative to normal weight. Model 1 was adjusted for age and sex; Model 2 was adjusted for school grade and sex and served as a sensitivity analysis. Multinomial models excluded children classified as underweight because of sparse cell counts. The overall association between assessment year and weight status in Model 1 was not statistically significant (likelihood-ratio test, *p* = 0.098). *p*-values were not adjusted for multiple testing. The nominally significant overweight contrast in Model 1 should be interpreted as exploratory rather than confirmatory.

**Table 5:** Multivariable binary logistic regression analysis of characteristics associated with membership in the 2024 cohort.

Variable	*B*	OR	95% CI	*p*-Value
Age	0.300	1.350	1.088–1.675	0.006 *
Sex (Girls vs. Boys)	−0.068	0.934	0.607–1.438	0.756
BMI	−0.045	0.956	0.898–1.018	0.161
KIDMED score	0.111	1.117	1.017–1.226	0.020 *
Overall self-concept	−2.371	0.093	0.051–0.170	<0.001 *

Legend: OR, odds ratio; CI, confidence interval; BMI, body mass index; KIDMED, Mediterranean Diet Quality Index for Children and Adolescents. The dependent variable was year of assessment (2018 = reference category; 2024 = outcome). Odds ratios were adjusted simultaneously for age, sex, BMI, KIDMED score, and overall self-concept. Sex was coded as girls versus boys. An asterisk (*) indicates nominal statistical significance (*p* < 0.05). *p*-values were not adjusted for multiple testing, and these exploratory associations should not be interpreted as confirmatory evidence.

**Table 6:** Multivariable binary logistic regression analysis of cohort membership including the five self-concept dimensions.

Variable	*B*	OR	95% CI	*p*-Value
Age	0.358	1.431	1.123–1.823	0.004 *
Sex (girls vs. boys)	0.040	1.041	0.638–1.700	0.872
BMI	−0.021	0.979	0.914–1.049	0.548
KIDMED score	0.130	1.139	1.025–1.265	0.015 *
Academic self-concept	0.388	1.474	0.961–2.262	0.075
Emotional self-concept	−0.585	0.557	0.421–0.736	<0.001 *
Social self-concept	−0.989	0.372	0.236–0.587	<0.001 *
Family self-concept	−1.553	0.212	0.132–0.340	<0.001 *
Physical self-concept	0.175	1.191	0.784–1.810	0.412

Legend: OR, odds ratio; CI, confidence interval; BMI, body mass index; KIDMED, Mediterranean Diet Quality Index for Children and Adolescents. The dependent variable was year of assessment (2018 = reference; 2024 = outcome). Odds ratios were adjusted simultaneously for age, sex, BMI, KIDMED score, and all five self-concept dimensions. Sex was coded as girls versus boys. An asterisk (*) indicates nominal statistical significance (*p* < 0.05). *p*-values were not adjusted for multiple testing, and these exploratory associations should not be interpreted as confirmatory evidence.

**Table 7:** Comparison of individual KIDMED questionnaire by assessment year.

KIDMED Item	2018 *n* (%)	2024 *n* (%)	Difference (pp)	95% CI	χ^2^	*p*-Value	Cramér’s *V*
Eats fruit every day	153 (81.4%)	188 (81.4%)	0.0	−7.4 to 7.6	0.000	1.000	0.000
Eats a second fruit daily	125 (66.5%)	147 (63.6%)	−2.9	−11.9 to 6.3	0.370	0.543	0.030
Fresh vegetables once daily	117 (62.2%)	153 (66.2%)	+4.0	−5.2 to 13.2	0.724	0.395	0.042
Eats vegetables more than once daily	100 (53.2%)	115 (49.8%)	−3.4	−12.9 to 6.2	0.482	0.488	0.034
Eats fish 2–3 times a week	114 (60.6%)	136 (58.9%)	−1.8	−11.1 to 7.7	0.134	0.714	0.018
Visits a fast-food restaurant >1/week	76 (40.4%)	62 (26.8%)	−13.6	−22.5 to −4.5	8.661	0.003	0.144
Eats legumes >1/week	109 (58.0%)	137 (59.3%)	+1.3	−8.1 to 10.7	0.075	0.784	0.013
Eats pasta or rice almost every day	105 (55.9%)	110 (47.6%)	−8.2	−17.6 to 1.4	2.811	0.094	0.082
Has breakfast cereals	145 (77.1%)	152 (65.8%)	−11.3	−19.7 to −2.6	6.443	0.011	0.124
Eats nuts regularly ≥2/week	108 (57.4%)	160 (69.3%)	+11.8	2.6 to 20.9	6.279	0.012	0.122
Use olive oil at home	157 (83.5%)	212 (91.8%)	+8.3	2.0 to 14.9	6.736	0.009	0.127
Skips breakfast	79 (42.0%)	80 (34.6%)	−7.4	−16.6 to 1.9	2.403	0.121	0.076
Dairy product for breakfast	114 (60.6%)	166 (71.9%)	+11.2	2.1 to 20.2	5.889	0.015	0.119
Commercial pastries for breakfast	69 (36.7%)	93 (40.3%)	+3.6	−5.8 to 12.7	0.553	0.457	0.036
Two yoghurts/40 g cheese daily	89 (47.3%)	81 (35.1%)	−12.3	−21.5 to −2.8	6.478	0.011	0.124
Sweets several times/day	65 (34.6%)	61 (26.4%)	−8.2	−17.0 to 0.7	3.288	0.070	0.089

Legend: CI, confidence interval; pp, percentage points. Frequencies refer to affirmative responses to each item. Between-cohort differences were calculated as 2024 minus 2018 and are expressed in percentage points, with 95% CIs calculated using Newcombe’s method based on Wilson score intervals. Comparisons used Pearson’s chi-square tests, with Cramér’s *V* as the effect-size measure. Confidence intervals and reported *p*-values are unadjusted for multiple comparisons; the Bonferroni-adjusted significance threshold was *p* < 0.003125 (0.05/16).

## Data Availability

The data that support the findings of this study are available from the corresponding author upon reasonable request. The data are not publicly available due to privacy and ethical restrictions relating to the protection and confidentiality of human-participant data.

## Supplementary Materials

The following supporting information can be downloaded at: https://www.mdpi.com/article/10.3390/healthcare14213697/s1, Table S1: Exploratory Year × Grade interactions and grade-specific between-cohort comparisons.

## Abbreviations

The following abbreviations are used in this manuscript:

BMI	Body mass index
PE	Physical Education
KIDMED	Mediterranean Diet Quality Index for Children and Adolescents
AF5	Form 5 Self-Concept Questionnaire
