# Supplementary material for: Mediterranean Diet Adherence, Weight Status, and Self-Concept in Primary Schoolchildren in Melilla, Spain: A Comparison of Two Independent Cohorts Assessed in 2018 and 2024
Source: Healthcare (Basel). 2026 Nov 1;14(21):3697. doi: 10.3390/healthcare14213697 (PMC13762662; doi:10.3390/healthcare14213697)
Supplement: Supplementary file 1 [file healthcare-14-03697-s001.zip › healthcare-4610428-supplementary.pdf]

Supplementary Table S1. Exploratory Year × Grade interactions and grade-specific between-cohort comparisons

Panel A. Year × Grade interaction tests

Outcome	F (2, 413)	*P*	Partial η^2
BMI	0.602	0.548	0.003
KIDMED score	2.373	0.094	0.011
Overall self-concept	5.629	0.004	0.027
Academic self-concept	3.552	0.030	0.017
Social self-concept	0.861	0.424	0.004
Emotional self-concept	4.597	0.011	0.022
Family self-concept	12.915	<0.001	0.059
Physical self-concept	4.168	0.016	0.020

Panel B. Grade-specific estimated marginal means and between-cohort contrasts

Variable / Grade		*n* (2018 / 2024)	2018 mean (95% CI)	2024 mean (95% CI)	Difference (95% CI)	Bonferroni-Adjusted *p*
**BMI**						
	Grade 4	57 / 79	18.381 (17.490–19.271)	18.149 (17.393–18.905)	−0.232 (−1.400, 0.936)	1.000
	Grade 5	62 / 79	19.804 (18.950–20.657)	19.630 (18.874–20.386)	−0.174 (−1.314, 0.967)	1.000
	Grade 6	69 / 73	19.344 (18.535–20.153)	19.917 (19.131–20.704)	+0.574 (−0.555, 1.702)	0.954
**KIDMED score**						
	Grade 4	57 / 79	5.632 (5.026–6.237)	5.671 (5.156–6.185)	+0.039 (−0.756, 0.834)	1.000
	Grade 5	62 / 79	6.161 (5.580–6.742)	7.101 (6.587–7.616)	+0.940 (0.164, 1.716)	0.054
	Grade 6	69 / 73	6.435 (5.884–6.985)	6.219 (5.684–6.754)	−0.216 (−0.984, 0.552)	1.000
**Overall self-concept**						
	Grade 4	57 / 79	3.793 (3.690–3.896)	3.563 (3.476–3.650)	−0.230 (−0.365, −0.095)	<0.003
	Grade 5	62 / 79	3.920 (3.822–4.019)	3.391 (3.304–3.478)	−0.530 (−0.661, −0.398)	<0.003
	Grade 6	69 / 73	3.873 (3.780–3.966)	3.589 (3.499–3.680)	−0.284 (−0.414, −0.153)	<0.003
**Academic self-concept**						
	Grade 4	57 / 79	4.170 (3.995–4.344)	3.943 (3.795–4.091)	−0.227 (−0.455, 0.002)	0.156
	Grade 5	62 / 79	3.892 (3.726–4.059)	3.829 (3.681–3.977)	−0.064 (−0.287, 0.159)	1.000
	Grade 6	69 / 73	3.952 (3.793–4.110)	4.151 (3.997–4.305)	+0.199 (−0.022, 0.420)	0.231
**Social self-concept**						
	Grade 4	57 / 79	3.924 (3.766–4.082)	3.507 (3.373–3.640)	−0.417 (−0.624, −0.211)	<0.003
	Grade 5	62 / 79	4.059 (3.908–4.210)	3.463 (3.329–3.596)	−0.597 (−0.798, −0.395)	<0.003
	Grade 6	69 / 73	4.017 (3.874–4.160)	3.568 (3.429–3.708)	−0.448 (−0.648, −0.249)	<0.003
**Emotional self-concept**						
	Grade 4	57 / 79	2.926 (2.696–3.156)	2.639 (2.444–2.834)	−0.287 (−0.589, 0.014)	0.186
	Grade 5	62 / 79	3.465 (3.244–3.685)	2.527 (2.332–2.723)	−0.937 (−1.232, −0.643)	<0.003
	Grade 6	69 / 73	3.374 (3.165–3.583)	2.740 (2.537–2.943)	−0.634 (−0.926, −0.343)	<0.003
**Family self-concept**						
	Grade 4	57 / 79	3.865 (3.721–4.010)	3.659 (3.536–3.782)	−0.207 (−0.396, −0.017)	0.099
	Grade 5	62 / 79	4.352 (4.214–4.491)	3.484 (3.361–3.606)	−0.868 (−1.054, −0.683)	<0.003
	Grade 6	69 / 73	4.309 (4.178–4.441)	3.613 (3.485–3.741)	−0.696 (−0.879, −0.513)	<0.003
**Physical self-concept**						
	Grade 4	57 / 79	3.991 (3.815–4.168)	3.948 (3.798–4.098)	−0.043 (−0.275, 0.189)	1.000
	Grade 5	62 / 79	3.898 (3.729–4.067)	3.499 (3.349–3.649)	−0.399 (−0.625, −0.173)	<0.003
	Grade 6	69 / 73	3.729 (3.569–3.890)	3.773 (3.617–3.929)	+0.043 (−0.180, 0.267)	1.000

Legend: BMI, body mass index; KIDMED, Mediterranean Diet Quality Index for Children and Adolescents; CI, confidence interval. Estimated marginal means were obtained from separate general linear models including assessment year, school grade, and their interaction. Differences were calculated as 2024 minus 2018. The 95% confidence intervals were not adjusted for multiple comparisons. Grade-specific p-values were adjusted using the Bonferroni method across the three grade-specific comparisons between 2018 and 2024 within each outcome (adjusted p = min[3 × unadjusted p, 1]). Interaction p-values were not adjusted across outcomes. All analyses were exploratory and should be interpreted cautiously given the number of outcomes and comparisons examined.
